# Structural Properties of [N1888][TFSI] Ionic Liquid: A Small Angle Neutron Scattering and Polarizable Molecular Dynamics Study

**DOI:** 10.1021/acs.jpcb.4c06255

**Published:** 2024-11-05

**Authors:** Shehan M. Parmar, William Dean, Changwoo Do, James F. Browning, Jeffrey M. Klein, Burcu E. Gurkan, Jesse G. McDaniel

**Affiliations:** † Department of Chemistry and Biochemistry, Georgia Institute of Technology, Atlanta, Georgia 30332, United States; ‡ Chemical and Biomolecular Engineering Department, Case Western Reserve University, Cleveland, Ohio 44106, United States; § Neutron Scattering Division, Oak Ridge National Laboratory, Oak Ridge, Tennessee 37831, United States; ∥ MPA-11: Materials Synthesis and Integrated Devices, Los Alamos National Laboratory, Los Alamos, New Mexico 87545, United States

## Abstract

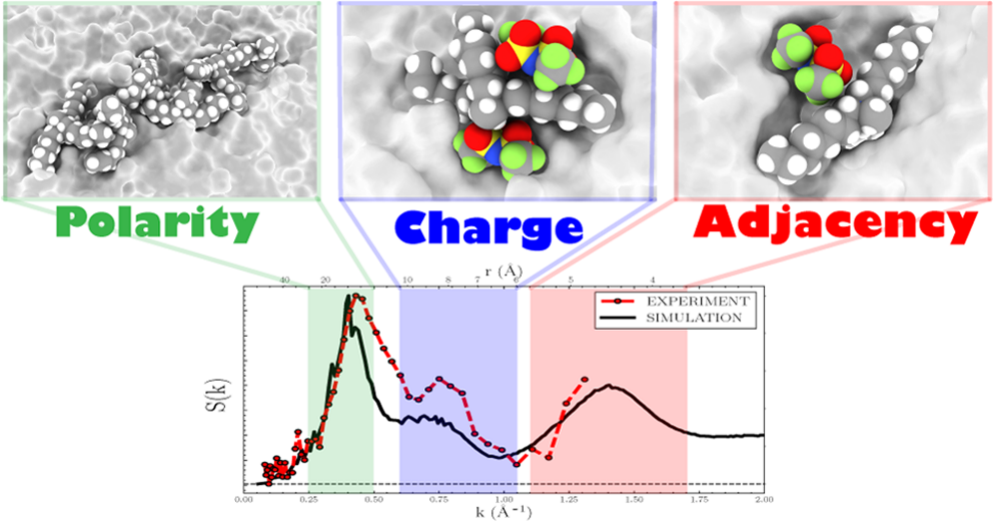

In this study, we investigate the quaternary ammonium-based ionic liquid (QAIL), methyltrioctylammonium bis(trifluoromethylsulfonyl)imide, [N_1888_][TFSI], utilizing small angle neutron scattering (SANS) measurements and polarizable molecular dynamics (MD) simulations to characterize the short- and long-range liquid structure. Scattering structure factors show signatures of three length scales in reciprocal space indicative of alternating polarity (*k* ∼ 0.44 Å^–1^), charge (*k* ∼ 0.75 Å^–1^), and neighboring or adjacent (*k* ∼ 1.46 Å^–1^) domains. Excellent agreement between simulation and experimental scattering structure factors validates various simulation analyses that provide detailed atomistic characterization of the different length scale correlations. The first solvation shell structure is illustrated by obtaining radial, angular, dihedral, and combined distribution functions, where two dominant spatial motifs, N^+^···N^–^ and N^+^···O^–^, compete for optimal packing around the polar head of the [N_1888_]^+^ cation. Intermediate and long-range structures are governed by the balance between local electroneutrality and octyl chain networking, respectively. By computing the charge-correlation structure factor, *S*_*ZZ*_, and the spatial extent of the octyl chain network using graph theory, the bulk-phase structure of [N_1888_][TFSI] is characterized in terms of electrostatic screening and apolar domain formation length scales.

## 1 Introduction

Ionic liquids (ILs) are multifaceted materials that have accelerated innovation in diverse domains, including carbon capture and sequestration (CCS),^1^ rare earth element (REE) extraction,^2,3^ water desalination,^4^ nanoparticle (NP) coating,^5^ rocket propulsion,^6^ and solar cell manufacturing.^7^ ILs are used as electrolytes in electrochemical applications and have garnered recent interest as replacements to typical organic electrolytes in lithium-ion batteries because of their numerous advantageous properties: broad electrochemical windows (>4 V),^8,9^ high thermal stabilities (250–400 °C),^10^ low vapor pressures, and nonflammability. Such macroscopic properties are a direct consequence of the fundamental electrostatic and structural characteristics. For example, strong intermolecular Coulombic attractive forces between cations and anions lead to large cohesive energies that dictate ion transport (i.e., high viscosity) and thermodynamics (i.e., low vapor pressure). More broadly, ILs have hypothetical tunability, where “targeted” molecular and compositional modifications—size and mass,^11−14^ side-chain length,^15−18^ conformational flexibility,^11,13,18−23,23,24^ charge asymmetry,^25−30^ chemical functionalization,^23,31−35^ mixture concentration and solvation^36−41^—can influence (and ideally control) thermophysical properties.^42^

The bulk-phase liquid structure plays an instrumental role in many of the mentioned applications of ILs. In electrochemical applications, the interfacial IL structure in addition to the bulk structure has an important influence on the system behavior. As a solvation shell transitions from the bulk to an interface, the ion packing is altered, and resulting electrical double layers (EDLs) form layers of alternating charge for up to several nanometers away from an electrode surface.^43−48^ Characterizing EDL formation and behavior for ILs is an ongoing research area.^46,49,50^ Notable features of the EDL, such as ion ordering and layering, applied voltage-dependence, or electrode-surface-dependence, are commonly explored by measuring the differential capacitance profile, , where σ is the surface charge density, and *U* is the applied voltage.^46,51−61^ Developing transferable EDL models across the diverse chemical composition space of ILs remains a significant challenge, however.^49,62,63^ Our present work is motivated by the importance of understanding the bulk-phase, structural characteristics of relatively complex ILs as a prerequisite for better understanding the interfacial structural properties of similar IL systems.^64,65^

Quaternary ammonium cation (QA)-based ILs (QAILs) make up one structurally unique class of ILs. QAILs have drawn a burgeoning interest due to their high thermal stabilities,^66^ high conductivities,^67^ and miscibility with a wide range of solvents^68,69^ while benefiting from lower cost and facile synthesis.^70^ In the context of electrochemical interfaces, QA cations provide enhanced EDL tunability through substantial structural variation, e.g., altering *C*_d_ profiles by changing alkyl chain length,^71,72^ and/or functionalization, e.g., stabilizing cathodic decomposition reactions via aliphatic moieties^67^ or improving miscibility via introducing hydroxyethyl moieties.^69^ Optimal tuning remains a challenge, however, due to the complex and broad-spanning bulk-phase structural behavior that QAILs encompass. Depending on the QA ion choice, the resulting bulk phase behavior can resemble typical room temperature ILs (RTILs), IL crystals (smectic, nematic, or columnar), or even ionic plastic crystals.^73^

QAILs are typically composed of tetraalkylammonium-based cations, N^+^R_4_, in which aliphatic or aromatic substituents, *R*, link to a positive nitrogen center; the length, symmetry, and type of the R group heavily influence physical properties and crystallinity.^73^ Just as the well-known ansatz, “If you want to understand function, study structure!”,^74^ defining features of QAILs are commonly explored through their bulk-phase, structural properties.^75^ In the case of aprotic, tetraalkylammonium-based ILs (i.e., trialkylmethylammonium, [N_1nnn_]^+^, for some alkyl chain length *n*), a limited number of experimental (e.g., neutron^76,77^ or X-ray scattering^78−84^) and molecular simulation^85−88^ studies have sparked debate regarding the nature of their short- and long-range ordering.

Pott and Méléard^79^ conducted one of the first systematic studies of [N_1nnn_]^+^ for *n* = 4, 6, and 8 with the common anion, bis(trifluoromethanesulfonyl)imide, or [TFSI]^−^. With corroborating molecular dynamics (MD) simulations,^88^ low wavevector “prepeaks” (*k* ≈ 0.4–0.6 Å^–1^) in static X-ray scattering structure factors, *S*(*k*), were reported as signatures of a “disordered smectic phase A”, which is indicative of interdigitated bilayers demarcated by polar and hydrophobic regions. These conclusions suggested that, ostensibly, [N_1nnn_]^+^-based ILs behave like 1-alkyl-3-methylimidazolium, [C_n_mim]^+^-based IL crystals with .^89−91^ However, upon further inspection by Santos et al.,^80^ experimental and computational analyses of temperature-dependent *S*(*k*) for [N_1444_][TFSI] showed no sign of mesoscopic ordering, but rather that, the low *k* prepeaks were more appropriately ascribed to the IL’s anisotropic solvation environment. Evidently, the challenge of interpreting low *k* peaks has necessitated numerous other investigations.^77,82,86,87,90,92^ Nonetheless, more recent efforts increasingly agree that ILs generally exhibit three disparate *S*(*k*) domains, where low, intermediate, and high *k* peaks highlight the existence of nanoscale structural heterogeneity from polar–apolar group alternation, positive–negative charge alternation, and adjacency of neighboring cation–anion pairs, respectively.^92,93^

In this work, we contribute to the ongoing discourse on long-range structure of [N_1nnn_]^+^-based QAILs by presenting a detailed structural analysis of the methyltrioctylammonium bis(trifluoromethylsulfonyl)imide, [N_1888_][TFSI]. The present study is partially motivated by recent experimental investigations of [N_1888_][TFSI] at electrochemical interfaces, for which there are open questions regarding the interfacial IL structure.^64,65,94^ For instance, Klein et al.^65^ posit two equally plausible, yet distinct, EDL models based on neutron reflectometry (NR) experiments for various solid–[N_1888_][TFSI] interfaces: the interfacial structure either (1) remains indistinguishable from the bulk on an unbiased surface or (2) contains one ion-rich layer of like-species (i.e., cations or anions) near the natively charged solid surface. In this work, we conduct small angle neutron scattering (SANS) experiments on the bulk [N_1888_][TFSI] to investigate the liquid structure at temperatures of 300, 330, 360, and 400 K. The neutron structure factors reported here complement X-ray structure factors that have been previously reported for [N_1888_][TFSI] IL.^79^ In addition, we perform molecular dynamics (MD) simulations utilizing an *ab initio*, polarizable force field to characterize short- and long-range coordination environments with atomistic resolution. The structure factors computed from the MD simulations are in excellent agreement with both the experimental neutron and the X-ray scattering data, validating the reliability of subsequent simulation analysis. From the simulations, we compute radial, angular, dihedral, and combined distribution functions to illustrate the ion structuring, as well as a novel analysis of structurally heterogeneous domains using graph theory.^95,96^

## 2 Methods

### 2.1 Small Angle Neutron Scattering

Small angle neutron scattering experiments were conducted on the EQ-SANS instrument at Spallation Neutron Source at Oak Ridge National Laboratory.^97,98^ Measurements were performed at a 1.3 m sample-to-detector distance using a wavelength band defined by a minimum wavelength of 1 Å, covering scattering wavevectors ranging from 0.07 to 1.5 Å^–1^. The sample was loaded in a 1 mm quartz cell and measured at 300, 330, 360, and 400 K. The obtained data were reduced after correcting for the detector sensitivity and subtracting background scatterings. The data were converted into absolute scale intensities (cm^–1^) using a porous silica standard sample.^97^ Methyltrioctylammonium bis(trifluoromethylsulfonyl)imide, [N_1888_][TFSI] (purity 99%), was purchased from Iolitec (Alabama, US) and used as received.

### 2.2 Molecular Dynamics Simulations

**Figure 1:**
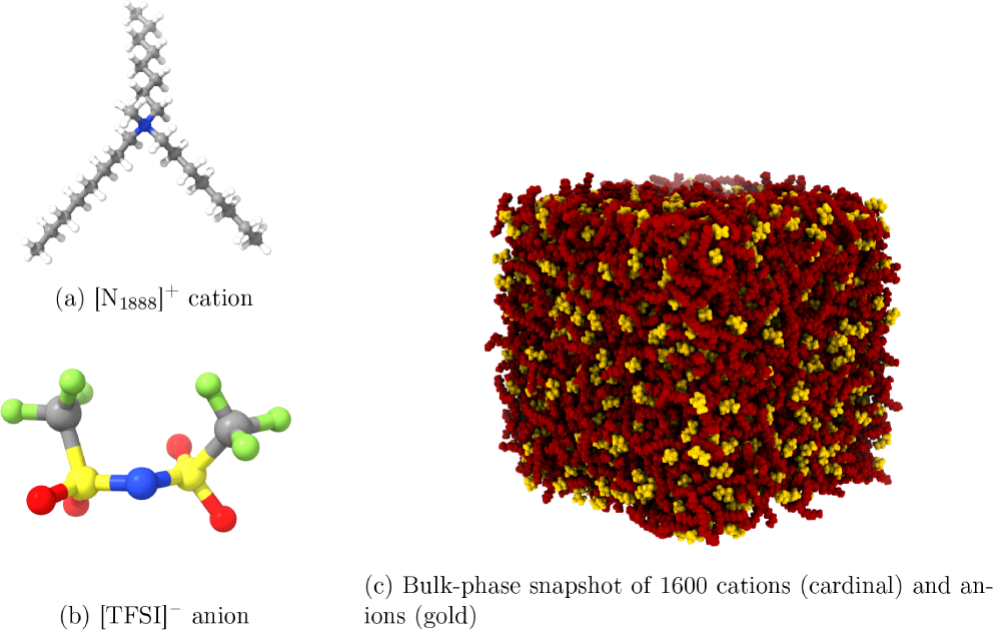
Molecular and bulk-phase structure of [N_1888_][TFSI] cation/anion pairs. Atoms include hydrogen (white), carbon (gray), oxygen (red), nitrogen (blue), sulfur (yellow), and fluorine (green).

We perform MD simulations of bulk [N_1888_][TFSI] IL in the NPT ensemble at 300, 400, 450, and 500 K temperatures and 1 bar pressure. The chemical structures of the ion pairs are shown in Figure 1a,b. The relatively long alkyl chains result in an overall, highly viscous (∼600 mPa s^64^) bulk liquid, requiring careful attention to statistical sampling in the simulations. Moreover, given the structurally complex and relatively bulky [N_1888_]^+^ cation (Figure 1a), we rigorously search for potential artifacts of finite simulation domain size, as discussed in Section 3.1. While Section 3.1 contains simulation benchmarks for a range of system sizes consisting of 200, 900, and 1600 ion pairs, unless otherwise stated, the remainder of the manuscript focuses on simulations of the 1600 ion pair system. Each simulation utilizes the previously developed SAPT-FF force field, which is an *ab initio*, polarizable atomistic model.^99,100^ Simulations are carried out with the OpenMM simulation software version 7.7.^101^ We utilize a dual-Langevin thermostat scheme^102^ for efficient treatment of Drude oscillators and set the friction coefficients for both thermostats to 1 ps^–1^; a Monte Carlo barostat was used for pressure coupling. Long-range electrostatics were computed using the particle mesh Ewald (PME) method,^103^ and van der Waals (VDW) interactions were truncated at 1.4 nm. All simulations were conducted on NVIDIA Tesla V100 GPUs for 100 ns to 1 μs of total sampling time at timesteps of 1 fs, with the longer trajectories for improved sampling at the lower temperature (e.g., 300 K); systems requiring greater sampling time were split up over several, separate MD trajectories as described in the Supporting Information. All simulation conditions are summarized in Table S1. All force field files and relevant scripts are given in the Supporting Information. A representative snapshot of the equilibrated ionic liquid system is shown in Figure 1c.

#### 2.2.1 Force Field Evaluation

To verify force field accuracy, a number of macroscopic properties were computed from ensemble averages of the simulation trajectory. The transferability of bond, angle, improper dihedral, and nonbonded terms for the SAPT-FF force field has been discussed in previous work;^99,100^ however, we further investigate the anion and cation proper dihedral angles as they are a key source of conformational flexibility and consequently affect bulk-phase behavior.^13,104^ As similar to other ILs,^100^ the population of TFSI anions is composed of ∼67/15% *cisoid* () and *transoid* () conformers, as shown by the “pseudo” dihedral distribution functions (DDFs) of ϕ_C–S–S–C_ in Figure S1. Note that this is a stark contrast from the *transoid*-dominated [N_1444_][TFSI] simulations conducted by Santos et al.^80^ and Lima et al!^82^ This behavior is rationalized by the influence of cation/anion ion-pair interactions, resulting in steric and packing constraints not present in the gas phase,^105^ as discussed at the length by McDaniel et al.^100^ Similarly, the C–C–C–C DDFs are composed of ∼20/75% *gauche* () and *trans* () conformers. This distribution is in semiquantitative agreement with quantum mechanical potential energy scans^106^ and numerous *n*-alkane conformational statistics in bulk liquids^107^ (e.g., liquid octane^108,109^)^.,^

Furthermore, the simulated density (1.126 g cm^–3^) shows excellent agreement within 2% of the experimental density (1.110 g cm^–3^).^79^ We also computed the liquid cohesive energies, *E*_coh_, which is a fundamental measure of how strongly bound the cations and anions are in the liquid. For neutral organic solvents, *E*_coh_ is directly related to the enthalpy of vaporization Δ*H*_vap_, but for ILs, such a comparison is considerably more complex due to ion pairing/association in the gas-phase;^99^ furthermore, we are not aware of experimental Δ*H*_vap_ data for the [N_1888_][TFSI] IL. As shown in Figure S2, the order-of-magnitude *E*_coh_ ∼ −550 kJ/mol and temperature-dependence are in qualitative agreement with common alkylimidazolium ILs benchmarked in previous work.^99^ Interestingly, the cohesive energy of [N_1888_][TFSI] is ∼50–100 kJ mol^–1^ larger in magnitude (per ion pair) than typical alkylimidazolium/BF_4_-based ILs: . This is consistent with the higher viscosity of [N_1888_][TFSI] compared to the alkylimidazolium ILs. The long alkyl side-chains of the [N_1888_]^+^ cation increase the van der Waals interactions (and thus cohesive energy) of the liquid; while a typical rule of thumb is that electrostatic interactions should decrease for larger molecular ions, in this case, the charge density of the [N_1888_]^+^ cation is very localized, and TFSI anions effectively pack around the positive nitrogen center (Section 3.4). Thus, the exact *E*_coh_ of [N_1888_][TFSI] comes from the subtle interplay between nonbonded interactions and the specific liquid structure.^42^ It is important to note, however, that this comparison of energy density *per ion pair* is not equivalent to comparison of *volumetric* energy density, due to the disparate sizes of the ions.

## 3 Results and Discussion

### 3.1 Finite-Size Effects

For computer simulations of complex liquids such as [N_1888_][TFSI], it is important to consider possible artifacts/finite-size effects introduced by the use of periodic simulation domains that are typically on the order of several to tens of nanometers. To circumvent larger and longer simulations, numerous corrective finite-size “scaling” methods have been developed that improve similar long-range asymptotic calculations (e.g., RDFs, Kirkwood–Buff integrals).^110−123^ However, finite-size scaling requires method-specific parametrization and is not necessarily transferable across all simulation analyses.^124^ Because [N_1888_][TFSI] is an electrolyte, its structural correlation functions are expected to satisfy macroscopic electrostatic screening conditions or sum rules.^125^ While early simulation studies of molten salts^126^ and ionic solutions^127^ emphasized the evaluation of sum rules as “an important test of the convergence of the electrolyte system to an equilibrium state”,^127^ to our knowledge, we report here the first example of applying sum rules to characterize finite-size effects in simulations of room-temperature ILs.

One unifying structural feature of ILs and molten salts is the anticorrelated nature of concentric coordination shells of cations and anions, which results from both charge neutrality and screening requirements.^128^ At a local, microscopic level, the electroneutrality condition gives rise to the following constraint on the pair-correlation or radial distribution functions,^125^
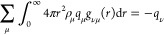
1where μ and ν are types of cations or anions,  is the ion type number density for *N*_ν_ number of ions within a volume *V* of species ν, *q*_ν_ is the charge of the ion, and  is the ion–ion radial distribution function (RDF). Effectively, eq 1 implies that the total charge cloud surrounding a central ion must be equal and opposite to the tagged ion’s charge, to ensure charge neutrality.

Electrostatic screening also markedly dictates the long-range behavior of the IL pair distribution functions. Being an electrolyte, the ionic liquid is expected to exhibit electrostatic screening at long-range, as quantified by the requirement on the dielectric response function (in Fourier-space) that . Within linear response theory, this screening requirement mandates conditions that the pairwise correlation functions of the IL must satisfy. These constraints are known as the Stillinger–Lovett sum rules, which when equivalently written in terms of the reciprocal space, structure factor, take the form^126−133^
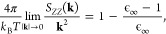
2where ϵ_∞_ is the infinite frequency dielectric response. *S*_*ZZ*_ is the charge-correlation structure factor given by,
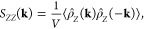
3where ⟨···⟩ denotes an ensemble average, **k** is the wavevector, and
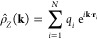
4is the Fourier component of the microscopic charge density, where *q*_*i*_ is the partial atomic charge, **r**_*i*_ is the position of the atom *i*, and *N* is the total number of atoms in the system. As the electrical susceptibility is proportional to *S_ZZ_*/*k*^2^,^125^ analysis of *S*_*ZZ*_ provides detailed insight about the electrostatic properties of the IL on microscopic length scales.^134^ Functionally, *S*_*ZZ*_ elucidates the length scales of the cation and anion correlations by weighting each pair correlation by the partial atomic charge, as shown in eq 4.^128^ We note that because the asymptotic limit of the charge correlation structure factor depends on ϵ_∞_, MD simulations with polarizable versus nonpolarizable force fields will fundamentally differ in their predicted ion structuring, as previously discussed in detail.^128^ In this work, all simulations employ a polarizable force field and thus ϵ_∞_ > 1, so that the right-hand side of eq 2 will be less than unity, as illustrated below.

**Figure 2:**
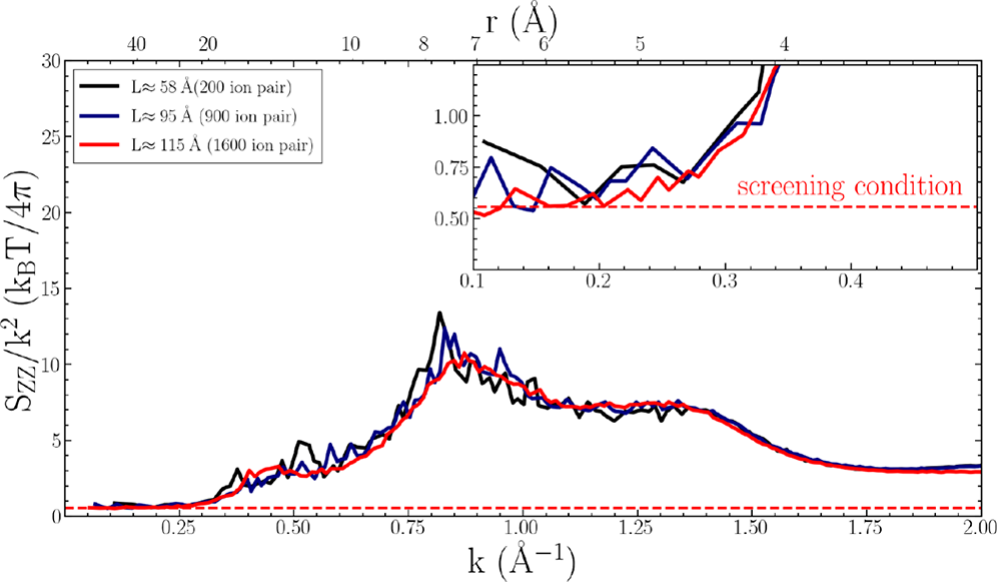
Charge-correlation structure factor *S*_*ZZ*_ for [N_1888_][TFSI] system sizes of length 58 Å (200 ion pairs, black curve), 95 Å (900 ion pairs, navy blue curve), and 115 Å (1600 ion pairs, red curve).

Verifying that eq 2 is satisfied within a computer simulation of an IL or any other electrolyte is an important test of equilibrium properties. Violation of the Stillinger–Lovett condition (eq 2) would imply either that the simulation provides insufficient statistics, is not at equilibrium, or is in a metastable state, or that substantial finite-size effects exist.^127^ To this end, we evaluated the charge-correlation structure factor from eq 3 for three equilibrated [N_1888_][TFSI] systems with different computational domain sizes consisting of 200, 900, and 1600 ion pairs. Leveraging particle-mesh Ewald (PME)^103^ algorithms, the structure factor *S*_*ZZ*_ was calculated directly in reciprocal space based on previously described methods.^128^ The resulting *S_ZZ_*/*k*^2^ curves are plotted in units *k*_B_*T*/4π for correspondence with eq 2, as shown in Figure 2 for the three different sized systems.

As will be discussed in further detail in later sections, the primary peaks in Figure 2 (*k* ∼ 0.85 Å^–1^) reflect length scales of real-space charge oscillations. However, the Stillinger–Lovett screening condition provides an important check on the validity of the predictions from these different system-size simulations. For various ILs (and indeed most liquids in general), the infinite frequency dielectric constant is approximately ϵ_∞_ ≈ 2,^133,135,136^ giving a value of ∼0.5 for the right-hand side of eq 2. As highlighted by the inset in Figure 2, all system sizes converge to the 1 – (ϵ_∞_ – 1)/ϵ_∞_ ≈ 0.5 asymptotic limit for typical ILs. The implications of mitigated finite size effects are significant—all subsequent static and structural analyses obey the charge sum rules and long-range electrostatic behavior of electrolytes. Nonetheless, the quantitative analysis of long-range correlations of such bulky ILs requires sufficient temporal and spatial statistics. Practically, the 1600 ion pair system provides enough of a length scale (*L*/2 ≈ 57.5 Å) to observe correlated behavior of the apolar domains, as will be shown in later sections. Due to these considerations, only simulation results for the large 1600 ion pair system will thus be discussed for the remainder of the paper.

### 3.2 Scattering Structure Factor Analysis

Like the functional form of eq 3, the X-ray and neutron scattering structure factors can be calculated from fluctuations of the microscopic *number* density,
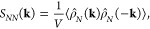
5where now microscopic number density  as a function of the **k** momentum transfer variable is defined based on the type of scattering. In the case of X-ray, the scattering amplitude is determined by elastic interaction with the electron density of a sample material, resulting in
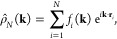
6such that the atomic form factor, , serves to “weight” the pairwise correlations observed in the scattering experiment. For scattering at small wavevectors as considered in this work, the **k**-dependence of  is typically small and  can be approximated by the atomic number. The asymptotic limit of the X-ray *S*_*NN*_ is
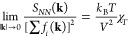
7where χ_*T*_ is the isothermal compressibility of the liquid.

Analogously, neutron *S*_*NN*_ is the result of elastic interactions with atomic nuclei, based on coherent scattering lengths. Thus, we calculate the scattering amplitude for the neutron structure factor by
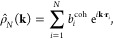
8where  is the coherent scattering length of the nucleus of atom *i*.^137^ Any incoherent scattering background is ignored in the neutron *S*_*NN*_ calculations, as it only adds a constant background as with the experimental measurements.

**Figure 3:**
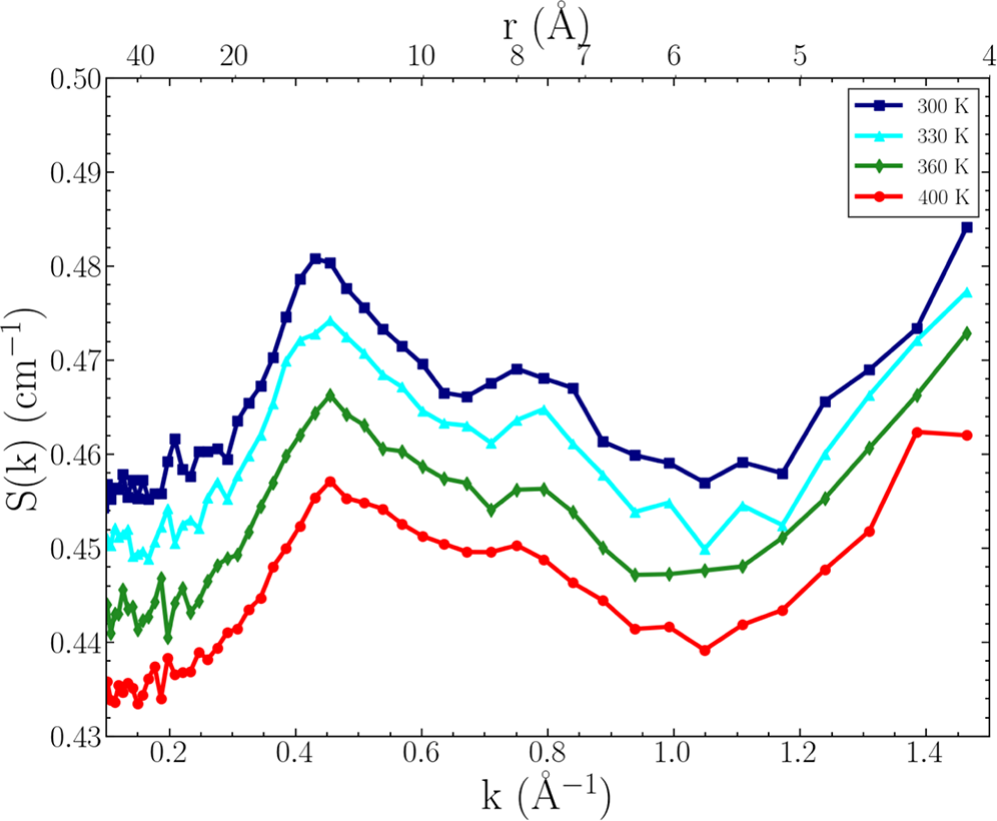
Temperature-dependent small angle neutron scattering (SANS) experimental measurements for [N_1888_][TFSI] ionic liquid.

Small angle neutron scattering (SANS) experiments were performed on the bulk [N_1888_][TFSI] IL at four different temperatures of 300 K, 330 K, 360 K, and 400 K. The results from the SANS experiments are shown in Figure 3. The scattering is governed by three characteristic local maxima, a prepeak at *k* ∼ 0.44 Å^–1^ followed by two peaks at *k ∼* 0.75 Å^–1^ and *k* ∼ 1.46 Å^–1^ within the *k* = 0.1–1.6 Å^–1^ range. Each peak is a feature of alternation within the bulk [N_1888_][TFSI], a behavior observed in common IL mixtures as well.^138^ At *k* ∼ 0.44 Å^–1^, the prepeak indicates the alternation of the polar (i.e., [TFSI] and the [N_1111_] ammonium head of [N_1888_]) and apolar (i.e., the octyl chains of [N_1888_]) groups, a feature that accounts for structural heterogeneity in a number of ILs.^77,93^ The strong peak at *k* ∼ 0.75 Å^–1^ reflects charge oscillation patterns between cations and anions, which is a general feature in molten salts and ILs, but the length scale for this feature is system dependent (ion size, structure, etc.).^125^ The final peak near *k* ∼ 1.46 Å^–1^ is attributed to correlations between adjacent atoms with strong Coulomb interactions. We follow the nomenclature by Araque et al.^93^ for each peak as the “polarity”, “charge”, and “adjacency” domain.

**Figure 4:**
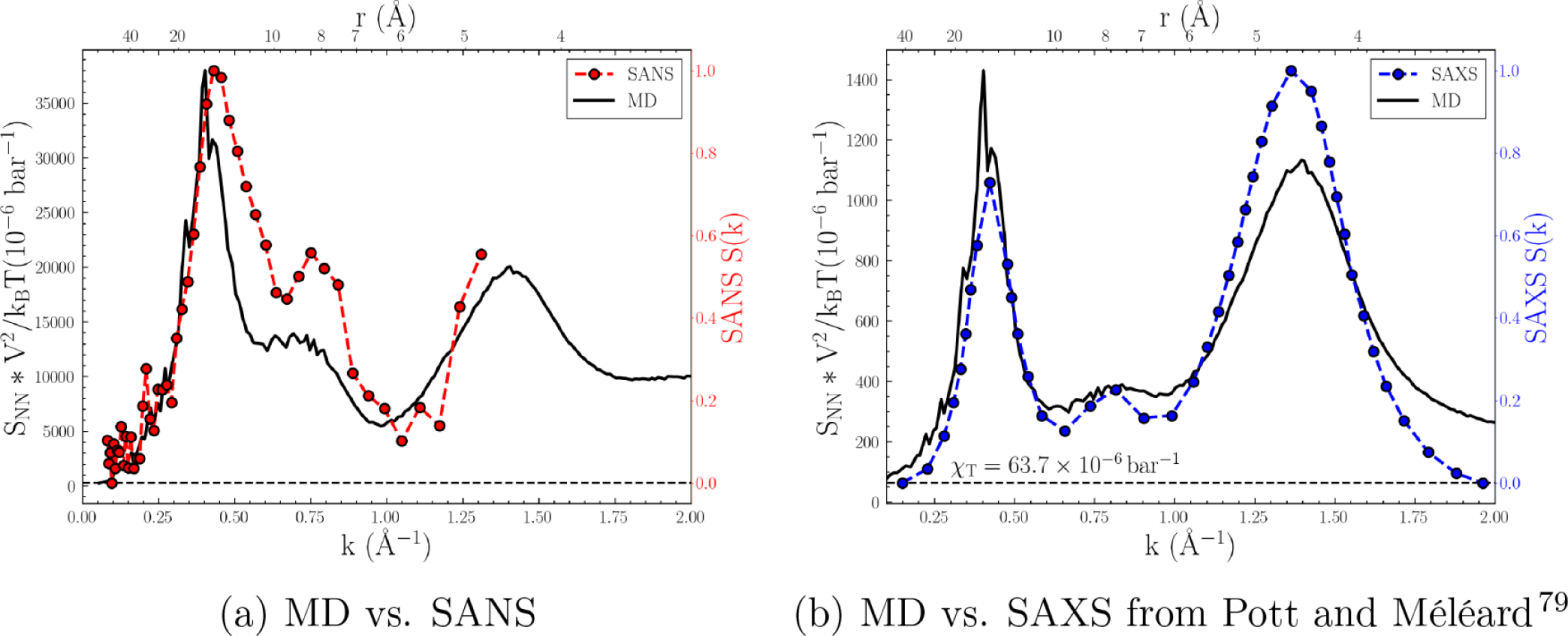
Scattering structure factor comparisons between MD simulations and (4a) small angle neutron scattering (SANS) measurements and (4b) small-angle X-ray scattering (SAXS) measurements at 300 K.

In Figure 4a, we compare the predicted neutron structure factor from the simulations to the experimental neutron scattering data (Figure 3) at 300 K. Additionally in Figure 4b, we compare the predicted X-ray structure factor to previous experimental, small-angle X-ray scattering (SAXS) data for [N_1888_][TFSI] reported by Pott and Méléard.^79^ It is observed that both neutron and X-ray *S*_*NN*_ comparisons show good agreement between simulations and experiments. The peak positions and relative error are provided in Table S2, showing ≤8% difference across all polarity, charge, and adjacency peaks. The computed isothermal compressibility from the X-ray *S*_*NN*_ asymptotic limit,  bar^–1^, is a reasonable value relative to that for commonly studied, imidazolium ILs,^128^ but to our knowledge, there is no existing experimental data for χ_*T*_ for the [N_1888_][TFSI] IL. We note that there is slightly greater error in the predicted neutron structure factor compared to that of the X-ray when compared to experimental data (≤8% for SANS and ≤5% for SAXS).

Figure S4 shows the temperature dependence of the neutron and X-ray structure factors predicted from the simulations and compared to the experimental data in Figure 3 as well as SAXS data from Pott and Méléard.^79^ Based on Figure S5, peak locations vary  between experiment and simulation. Like Santos et al. observed,^80^Figure S5 shows that at increasing length scales (or decreasing wavevectors *k*), variations in peak position as a function of temperature decrease. However, without any clear monotonic trends, as may be generally expected, no definitive conclusions are made regarding the temperature dependence. Santos et al.^80^ claim a similar conclusion, albeit at a 185–351 K range for small- and wide- angle X-ray scattering (SAXS-WAXS) measurements, where “peak shifts are likely to be a simple consequence of density changes in a material that has not undergone a first order transition.”

We also highlight the importance and complementary role of comparing SAXS and SANS experiments together. Lo Celso et al.^77^ showed that some neutron diffraction features, namely the initial low-*k* prepeak, can go undetected in SAXS measurements for certain ILs. However, the consistent prepeak–peak–peak pattern across both experimental studies shows strong evidence for structural heterogeneity due to polarity alternation in [N_1888_][TFSI]. Interestingly, in [N_1444_][TFSI], this feature was consistently missing in multiple studies,^79,80,82^ suggesting that the octyl chain length (∼10–11 Å) surpasses the threshold necessary for coordination of the apolar domains. We explore this hypothesis in further detail in later sections.

### 3.3 Partial Structure Factors

**Figure 5:**
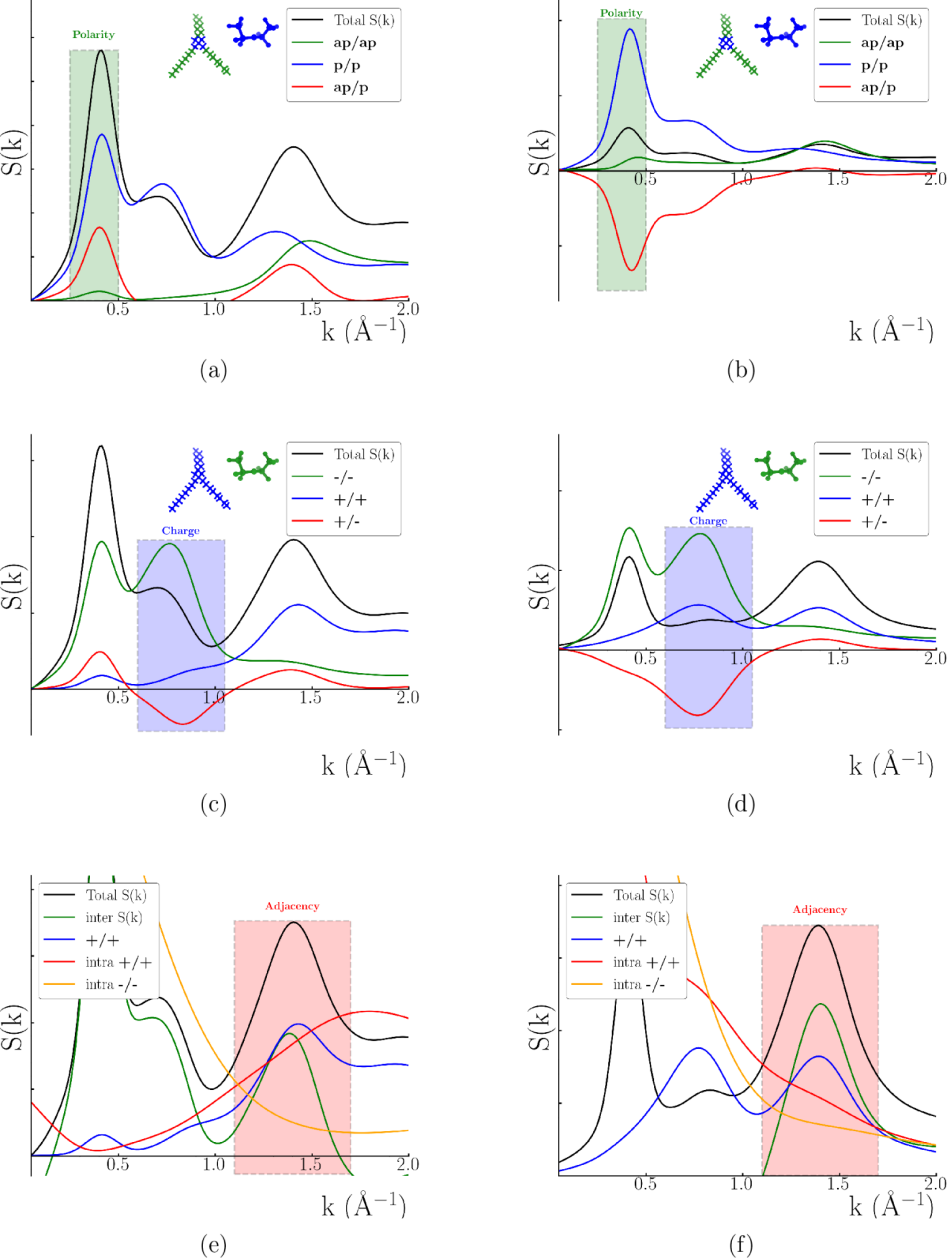
Partial neutron (left column, a, c, e) and X-ray (right column, b, d, f) scattering structure factors based on polarity (a, b), charge (c, d), and adjacency (e, f) domains. For Figures 5a–d, the colors correspond to the color of the polar/apolar or cation/anion group highlighted in the inset; the adjacency domain subfigures show the total (black), intermolecular (green), cation–cation (blue), and intramolecular anion (yellow) and cation (red) components of the structure factor.

Unlike in experimental studies, the scattering structure factors computed from MD simulations can be further decomposed into partial structure factors and analyzed within wavevector-dependent partitions that elucidate the source of short- and long-range correlations. The nature of eqs 4, 6, and 8 enable partitioning schemes for the polarity,^93^ charge,^128^ and adjacency^80^ domains as summarized in eqs S1–S7. In Figure 5, the total neutron (a, c, e) and X-ray (b, d, f) *S*_*NN*_ are shown with their respective partial structure factors. For ease of comparison, each partial structure factor was computed without the squared normalization term (e.g., as in eq 7).

#### 3.3.1 Polarity Domain

The low-**k** prepeak (*k* ∼ 0.44 Å^–1^) is investigated via contributions from the polar–polar, polar–apolar, and apolar–apolar interactions present in [N_1888_][TFSI], as shown in Figure 5a,b. As highlighted in the inset, the polar components consist of the entire [TFSI] anion and cationic head, CH_3_N(CH_2_)_3_, while the apolar components consist of the rest of the cation tail in the octyl chain. In both the neutron and X-ray *S*_*NN*_, the low-**k** prepeak is unambiguously dominated by the polar–polar contributions. The polar–polar and apolar–polar peak–antipeak behavior observed in the X-ray *S*_*NN*_ has been previously referred to as the “hallmark” of alternations present among “opposite-type” species.^93^ Thus, the peak–antipeak behavior illustrates an important structural feature, where a polar, cationic head–anion network alternates with the apolar octyl chains. The difference in relative peak heights between neutron and X-ray *S*_*NN*_ is accounted for by the different elemental contributions to the scattering intensity, i.e., eqs 6 and 8. Namely, the partial X-ray *S*_*NN*_ show a greater sensitivity to the high electronic density and consequently, the atomic form factors, of the TFSI anions (i.e., the “reporters of structure”^92^) than the neutron coherent scattering length scales.

#### 3.3.2 Charge Domain

The intermediate peak (*k* ∼ 0.75 Å^–1^) is similarly investigated via contributions from the cation–cation, cation–anion, and anion–anion interactions. The total *S*_*NN*_ show relatively subtle charge domain peaks (i.e., visibly a shoulder in Figure 4a and the smallest of all peaks in Figure 4b), a commonly observed feature often explained by complex interference cancellations.^80,139−142^ In reality, Figure 5c,d indicates definitive charge alternation based on the anion–anion and cation–anion peak–antipeak behavior, respectively. From the partial structure factor analysis, it is concluded that the anion–anion correlations are a substantial contribution to the peak (*k* ∼ 0.75 Å^–1^) in the charge domain region; this is consistent with analysis of other ILs.^128^ As will be discussed later, charge domain partitioning uncovers the fundamental length scale of cation–anion Coulomb interactions that align with the charge-correlation structure factor, *S*_*ZZ*_.

#### 3.3.3 Adjacency Domain

Lastly, the final peak at *k* ∼ 1.46 Å^–1^ can be investigated in terms of the inter- and intramolecular interactions present in the bulk-phase IL. A complimentary insight unveiled by the charge domain partitioning is the relatively large cation–cation *S*(*k*) contribution toward the high-**k** adjacency peak, both functionally and in relative weight, as shown in Figure 5e,f. From eq S6, the cation–cation *S*(*k*) can be simply decomposed into its relative inter- and intramolecular contributions—as shown by the red curves in Figure 5e,f, the intramolecular components were derived by sampling single molecular conformations from bulk phase trajectories, recomputing the structure factor, and scaling by the number of ions. The anion–anion intramolecular components contribute a nearly uniform background signal to the overall adjacency peak. Between the neutron and X-ray *S*_*NN*_, it is clear that while the intramolecular component adds a nontrivial contribution to the overall adjacency peak height, the *total* intermolecular components (including cation–anion cross terms) still remain the source of the *k* ∼ 1.46 Å^–1^ peak. This result is comparable to small- and wide-angle X-ray scattering and inter/intramolecular contribution comparisons for [N_1444_][TFSI] presented by Santos et al.,^142^ where intramolecular contributions only began to align with total scattering functions after *k* > 3 Å^–1^.

### 3.4 Domains Analysis

#### 3.4.1 Real-Space Adjacency Correlation Analysis

**Figure 6:**
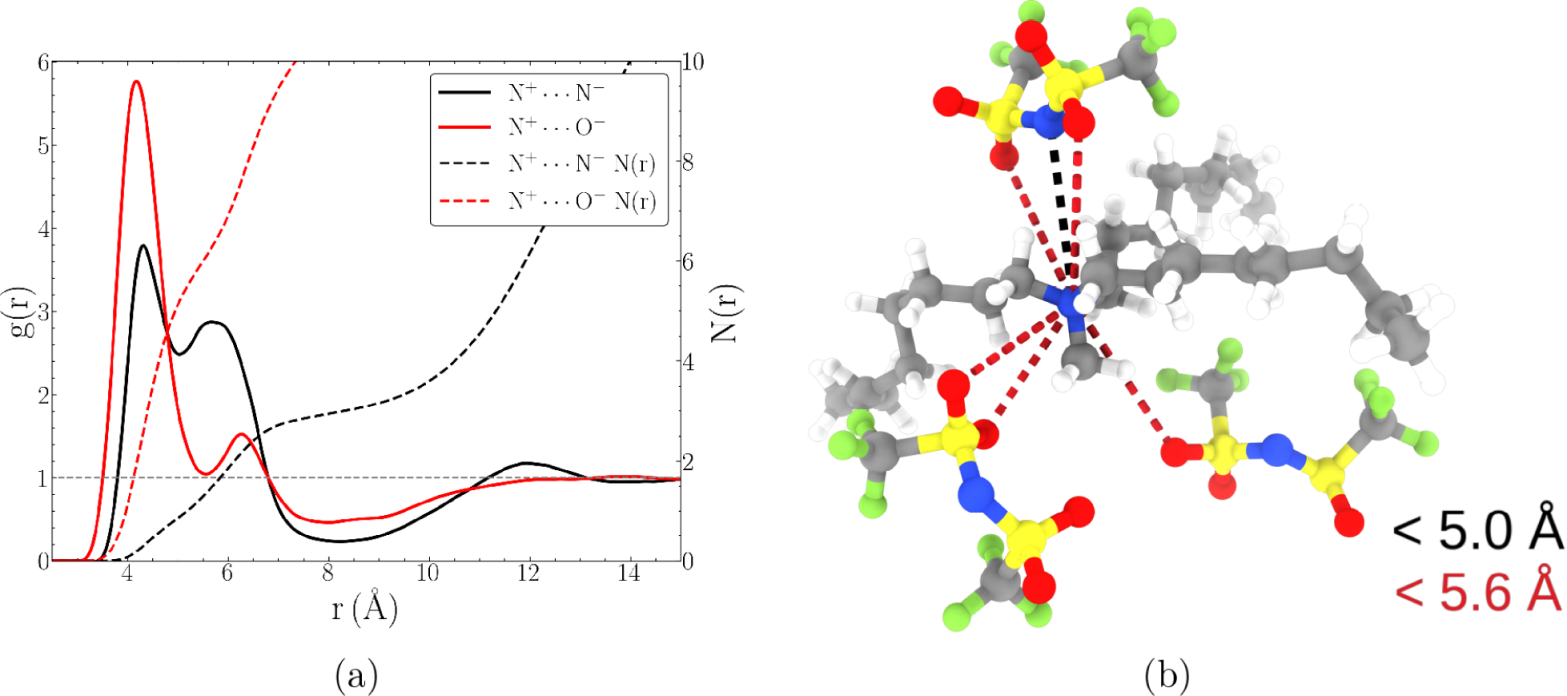
(a) Atomistic radial distribution function (solid lines) and corresponding running coordination number (dashed lines) for N^+^···N^–^ (black) and N^+^···O^–^ (red) coordination sites. (b) Representative snapshot of first solvation shell consisting of three coordinated anions to single cation; black and red lines highlight coordinated N^–^ and O^–^ atoms coordinated with cationic nitrogen, N^+^ within approximate first solvation shell radius, respectively; corresponding 360 deg video provided in the Supporting Information.

The structural signatures encoded by the three peaks in the structure factors can be further investigated via real-space analyses, as done in the ensuing sections. The adjacency domain in real-space can be investigated by the radial distribution functions (RDFs), shown in Figure 6a. The peak heights of the N^+^···O^–^ and N^+^···N^–^ are located at  = 4.18 Å and  = 4.33 Å, respectively. This length scale aligns remarkably with the adjacency peaks in Figure 4, 2π/1.46 ≈ 4.30 Å. The RDF peaks also highlight two underlining motifs where the [TFSI] anion is facing the cation through the N^–^ or O^–^, as shown in Figure 6b. The relative heights of the peaks indicate that the N^+^···O^–^ coordination site is more favorable than the N^+^···N^–^. The running coordination numbers, *N*(*r*), provide further insight into the coordination environment within the first solvation shell of the cation–anion pairs. We compute the *N*(*r*) from eq 9,
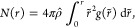
9where  is the average number density of the observed, anion atoms,  and . At the first local minima of the RDFs, the competition of the two motifs is clearly quantified by  as opposed to . The second local minima are ancillary; the secondary peaks are simply induced by the motifs in the first peaks by the alternate atom on the same molecule.

**Figure 7:**
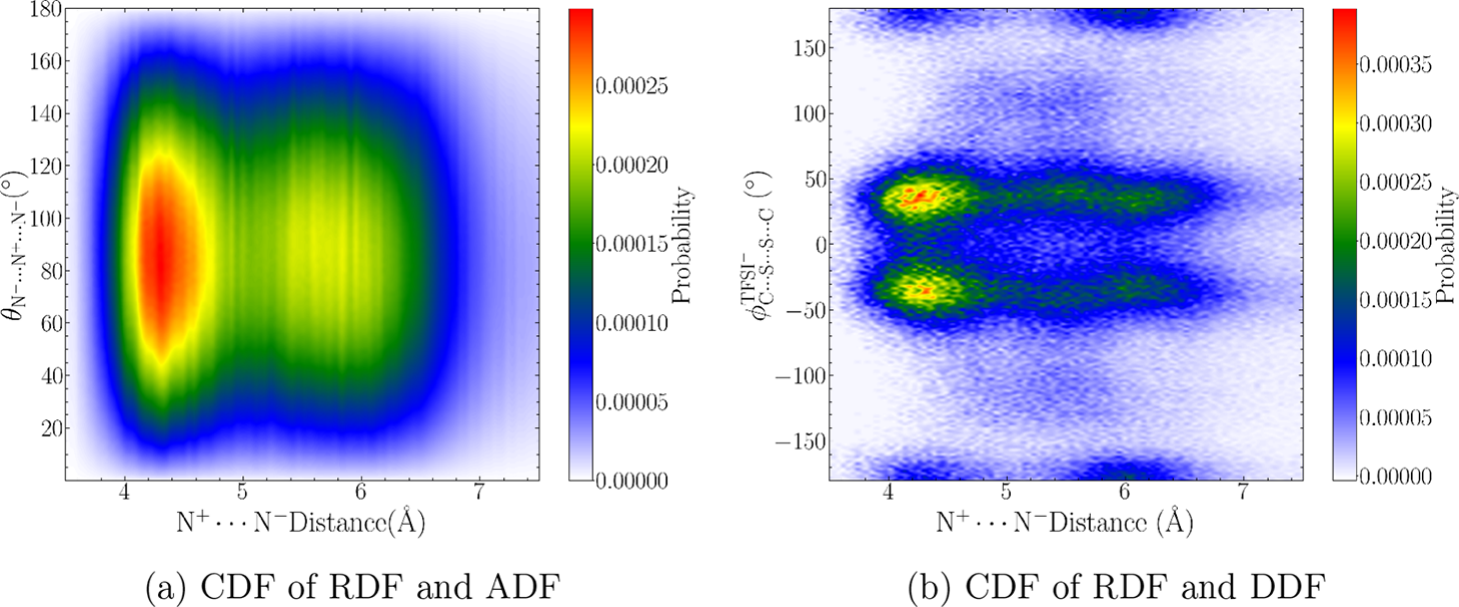
Combined distribution functions (CDFs) of radial distribution functions (RDFs) with (a) angular and (b) dihedral distribution functions.

While the RDFs illustrate the connectivity of pairwise coordination, the intricate detail of the first solvation shell may be further elucidated with angular and dihedral distribution functions (ADFs and DDFs, respectively). However, ADFs and DDFs alone do not necessarily preserve the symmetries recovered from the RDFs—thus, we apply combined distribution functions (CDFs), which have shown to be a powerful tool to illustrate ion coordination using the atomistic detail provided by computer simulations.^143^ Concretely, Figure 7 shows a two-dimensional probability density of pairwise N^+^···N^–^ distances binned with N^–^···N^+^···N^–^ angles (Figure 7a) and proper C···S···S···C pseudodihedral angles (Figure 7b). The N^+^···N^–^ motif optimally packs within the first solvation shell by forming an angle of 60° < θ < 120° with respect to a neighboring N^+^···N^–^ coordination site. Just as corroborated by the snapshot in Figure 6b, anions pack within the bisector defined by the octyl chains of the [N_1888_]^+^ cation, a similar conclusion made by Lima et al.^82^ for a [N_14444_]^+^-based system. Moreover, Figure 7b indicates that the first solvation shell is predominantly composed of *cisoid* conformations based on the high density of ±45° anions at the corresponding first peak in the RDF.

**Figure 8:**
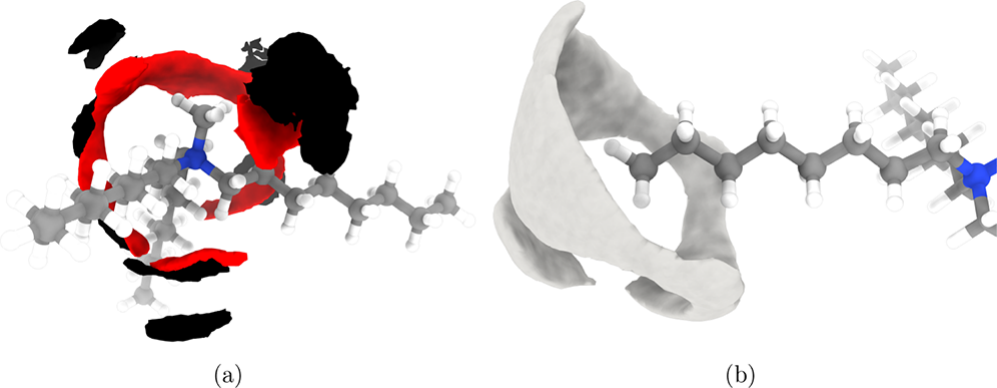
Spatial distribution functions (SDFs) of (a)  (solid red) and N^+^···N^–^ (solid black) coordination sites at 11.3 nm^–3^ and (b) terminal carbon of one single octyl chain with every other carbon (e.g., C_8_···C_1–25_) at 1.7 nm^–3^. Complete 360° videos are included in the Supporting Information.

Finally, we show the three-dimensional, spatial distribution function (SDF) calculated in TRAVIS^144^ to fully illustrate the short-range cation–anion packing. As shown in Figure 8a, the solid red () and solid black (N^+^···N^–^) isosurfaces highlight how the anion preferentially occupies the space between apolar octyl chains. When plotted at the same contour level (11.3 nm^–3^), the SDF isosurfaces clearly show an increase in surface of red,  “blobs” within the first solvation shell (∼102 Å^–2^) compared to the black (∼63 Å^2^). The 360° video included in the Supporting Information shows apparent overlap of red and black isosurfaces and, thus, similarity in the local position of the anion nitrogens and oxygens. Additionally, the SDF in Figure 8b provides a local perspective of cation–cation coordination. Based on the intercation carbon–carbon RDFs (Figure S7), the most apparent structuring occurs at the tail of the octyl chains; to this end, a single terminal carbon (e.g., C_8_) was selected as a reference atom and the subsequent SDF was computed for all possible terminal-carbon–carbon coordination sites (C_8_···C_1–25_). The morphological difference between the cation–anion (Figure 8a) and cation–cation (Figure 8b) SDF isosurfaces corroborates the “punctured sphere” versus “sleeve-like” spatial arrangement of the polar and apolar networks, respectively.^88^ However, as we will further investigate in Section 3.4.3, understanding structural nanosegregation requires analysis of apolar domain coordination *globally,* i.e., the *local* description of cation–cation coordination provided by an SDF manifests in long-range correlations.

#### 3.4.2 Charge Alternation Analysis

**Figure 9:**
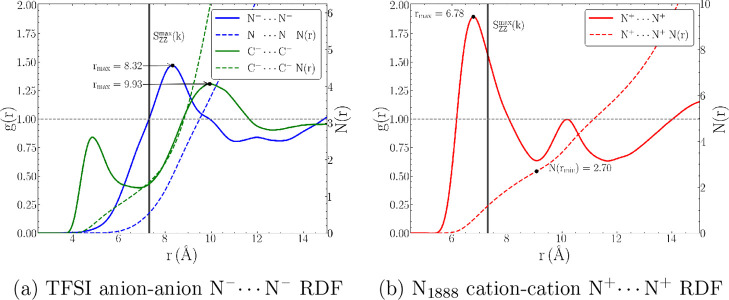
Radial distribution functions (RDFs) for like-species coordination, including (a) anion–anion and (b) cation–cation pairwise interactions.

Beyond the first solvation shell, the intermediate and long-range structure is governed by the charge sum rules and local electroneutrality in ILs.^125^ While the intermediate wavevector peak (*k* ∼ 0.75 Å^–1^) in neutron and X-ray scattering factors are typically subtle or even missing, the charge correlation structure factor *S*_*ZZ*_ peak (eq 5) (as was shown in Figure 2) shows a sharp peak near the reciprocal space length scale, ∼0.85 Å^–1^ at 300 K. Equivalently, in real space, anion–anion (Figure 9a) and cation–cation (Figure 9b) RDFs show peaks within ∼7–10 Å in real-space, as shown in Figure 9 with the *S*_*ZZ*_ peak shown as a solid, vertical line. The atomistic RDFs involving localized negative and positive charges, N^–^···N^–^ and N^+^···N^+^, respectively, span ∼7–8 Å and show excellent agreement with the *S*_*ZZ*_ peak. In previous work, this peak has been shown to identify the charge alternation length scales of common ILs,^128^ and for comparative purposes, Figure S6 shows the *S*_*ZZ*_ of [N_1888_][TFSI] (peak of ∼0.85 Å^–1^) compared to that of the prototypical, 1-butyl-3-methylimidazolium tetrafluoroborate [BMIM][BF_4_] IL (peak of ∼1 Å^–1^). We also include the C^–^···C^–^ (green curve in Figure 9a) as another measure of anion packing. The C^–^···C^–^ exhibits a typical bifurcated peak found in the literature,^128,145,146^ where a peak at ∼5 Å indicates presence of hydrophobic domains on the anion (i.e., CF_3_) and ∼10 Å indicates longer-range coordination length scales.

Consequently, two conclusions are made evident. First, the scattering structure factor peaks, either *S*_*NN*_ or *S*_*ZZ*_, *directly* correspond to the distance between successive coordination shells (i.e., with respect to some central ion) based on like-species (i.e., cation–cation and anion–anion) coordination distances. Second, compared to the common IL, [BMIM][BF_4_], there is a clear shift toward larger like-species distances due to the presence of apolar octyl chains that displace charged species for favorable long-range packing.

#### 3.4.3 Polarity Alternation Analysis

As was highlighted in the Introduction, [N_1888_][TFSI] is a unique IL in that it exhibits both typical charge alternation domains but also long-range ordering based on low-**k** scattering peaks. However, the degree of ordering is often difficult to interpret from scattering peaks alone and has evidently warranted much discussion and controversy in the literature. For example, low-**k** peaks similar in intensity and location have been observed in ILs that span a wide-range of phase behavior, anywhere from isotropic, smectic mesophase to near-crystalline or glass-forming liquids.^75^ In the case of TFSI-based ILs specifically, much insight can be drawn from the vast body of X-ray^78,81,147−151^ and neutron^152,153^ scattering studies (e.g., alkyl chain length increases ordering, hydroxyl-substitutions decreases ordering, etc.). Nonetheless, interpreting phase-behavior from scattering intensities alone is challenging, given its sensitive nature to the anion electronic structure.^93^ To this end, we invoke a theoretical, graph-based analysis to quantify the spatial extent of the apolar, octyl-chain network, similar to previous studies.^96^

Only until recently have theoretical, graph-based analyses been used to analyze MD trajectories. For instance, Lee et al.^95^ explored graph-theoretical approaches to understand ion aggregation morphology of various salt-based solutions. Recently, Stoppleman and McDaniel^96^ explored the spatial extent of hydrogen bond networks by establishing edges between neighboring water molecules (nodes) based on O···H distance and O···H···O angle criteria. We build on this work to understand the spatial extent of octyl chains within the overarching molecular simulation domain. In doing so, we define a graph  composed of molecular nodes  and criteria-based edges, . In each frame of the MD trajectory, we search all [N_1888_]^+^ cations (within a cutoff) that neighbor each other by two, tunable critical parameters: minimum interatomic carbon–carbon distance, , and the minimum number of such coordination sites, *n*. To determine , we compute all combinations of the atomistic, carbon–carbon RDFs and report a select few in Figure S7. In Santos et al.,^80^ it was sufficient to report such an RDF to discount the interdigitated bilayer hypothesis specifically for [N_1444_][TFSI] presented by Pott and Méléard.^79^ However, we use this RDF as a means to bound the  criterion in determining the overall octyl-chain graph network. We tune *n* by expecting an upper bound of 24—i.e., all octyl chains are interdigitated—and lower bound of at least one. For each frame in the MD trajectory, we compute the diameters, *d*, of all disjoint subgraphs, , defined as:
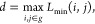
10where  is the minimum distance (in number of nodes) between nodes *i* and *j* for all .

**Figure 10:**
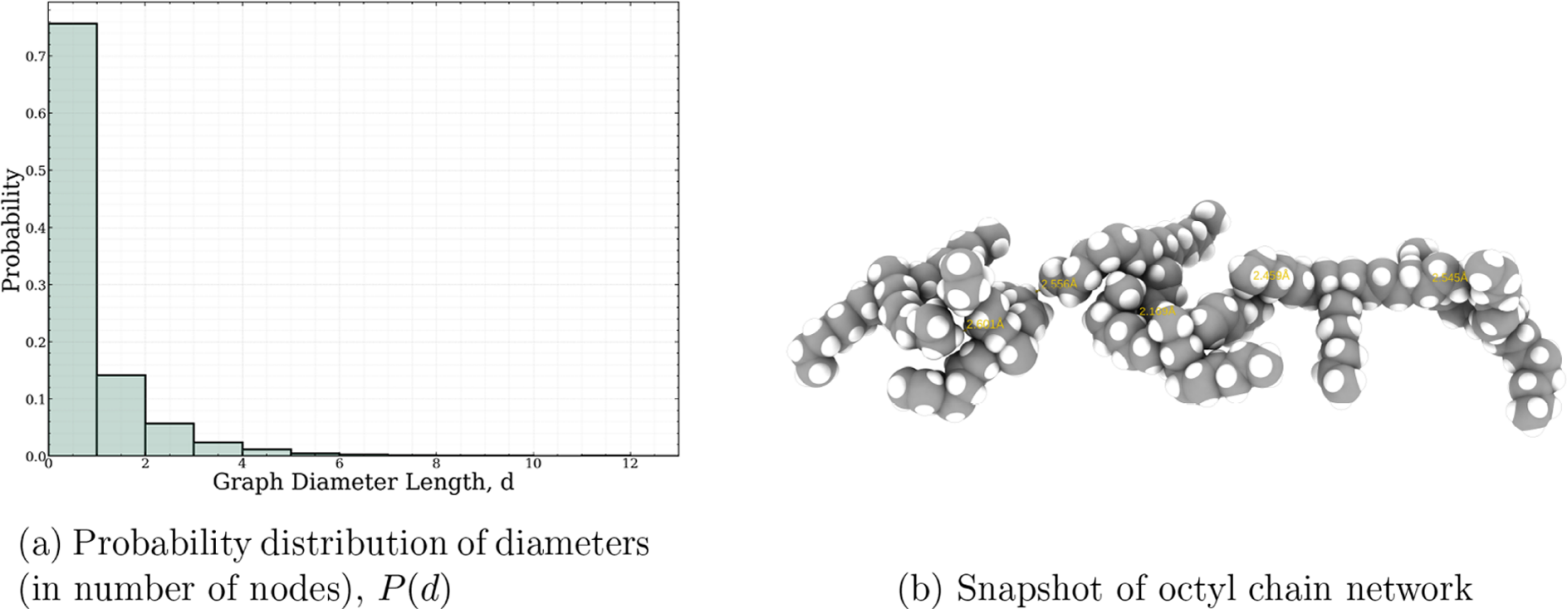
Probability distribution of (10a) octyl chain network diameters and (10b) visual snapshot of six [N_1888_]^+^ cations connected via distance criterion.

The octyl chain network is then characterized by the probability distribution for *g* to have a certain diameter, *P*(*d*). Parameter tuning was explored for  and ; as expected, extreme ends of the parameter space () show very noisy and limited (if any) statistics across the range of diameters that span the length of the simulation box size, 115 Å. Figure 10a thus illustrates the most representative behavior of *P*(*d*) for a selected  and *n* = 1. While small probabilities of octyl chains appear in apolar networks that span up to six subsequent octyl chains (e.g., Figure 10b), the predominant long-range motif is evident by the large peak at the *d* = 1 node. By this measure, the apparent low-**k** peak from Figure 4 can be interpreted as ∼2–3 neighboring [N_1888_]^+^ cations correlated by their octyl chains.

So then why, despite its large, bulky molecular structure, does [N_1888_][TFSI] more so resemble an isotropic liquid than a liquid-crystalline material? Between a visual inspection from Figure 1c and quantitative analysis from Figure 10a, it is clear that nanoscale spatial heterogeneities exist; however, the degree of ordering does not lead to “orientational and/or positional long-range order in at least one direction” (e.g., periodic stacking of molecular layers), a requisite for ionic liquid crystal (ILC) or mesomorphic behavior.^154^ The answer is 2-fold, stemming from (1) the subtle balance between the relative apolar to polar volume, i.e., *V*_alkyl_: *V*_polar_(155) and (2) the crucial importance of electrostatic screening. It is known that larger *V*_alkyl_: *V*_polar_ ratios induce higher degrees of nanosegregation of charged and uncharged domains, leading to highly ordered systems and smectic behavior.^89,154^ In the case of [N_1888_][TFSI], on one hand, the relatively large TFSI anion can generally inhibit long-range ordering due to its charge delocalization and increased configurational entropy from the low N–S torsional energy barrier.^156^ As an example, the study of several 1-methyl-3-(*n*-alkyl)imidazolium ([C_nMIM_]) [TFSI]^−^-based salts shows liquid crystal (LC) behavior only after the alkyl chain length was increased to *n* = 22.^154^ Moreover, Goossens et al.^154^ highlight that variations in *V*_alkyl_: *V*_polar_ can “only show a smectic mesophase if the ionic headgroups and the alkyl chains are able to project comparable cross-sectional areas onto the ionic sublayer planes. As such, the salts with three equivalent long *n*-alkyl chains require larger anions than the corresponding salts with only two long *n*-alkyl chains to exhibit a smectic LC phase.”

## 4 Conclusion

We present a joint simulation and experimental study of a quaternary ammonium-based IL, [N_1888_][TFSI], to comprehensively characterize its bulk-phase structure. Small angle neutron scattering (SANS) experiments were conducted for a range of temperatures, and the measured structure factors were utilized to validate simulation predictions; the predicted structure factors show excellent agreement between simulation and the experiment data, as well as previously published small-angle X-ray scattering (SAXS) data.^79^ By partitioning the computed scattering structure factors by polar/apolar, cation/anion, and inter/intramolecular components, the reciprocal-space results indicate alternation of polarity (*k* ∼ 0.44 Å^–1^), charge (*k* ∼ 0.75 Å^–1^), and adjacency (*k* ∼ 1.46 Å^–1^) domains.

Moreover, we explore each domain via further real-space analyses. For instance, the radial distribution functions (RDFs) highlight two spatial motifs where the anion nitrogen and oxygen competitively pack around the cationic polar head. Combined distribution functions (CDFs) complete the analysis of the first solvation shell by elucidating the favorable packing of anions (1) within the bisector of the cation octyl chains and (2) in the *cisoid* conformation. At the intermediate range, the charge correlation structure factor, *S*_*ZZ*_, recovers the exact length scale of the distance between subsequent solvation shells that manifest from charge oscillations. At long range, we employ a novel, graph network analysis to conclude the spatial extent of coordinated cations spanning two to three molecules, enough to show signatures of nanoscale heterogeneity but still resemble an isotropic IL.

Quaternary ammonium-based ionic liquids (QAILs) resemble a sandbox for tuning the chemical properties at electrochemical interfaces. In practice, however, their fundamental behaviors in charged and dynamic environments are inescapably linked to the static, bulk-phase liquid structure. In future work, we aim to explore the pertinent question, how does voltage modulate the electrical double layer of [N_1888_][TFSI] at charged interfaces? The definitive understanding of motifs and long-range phase behavior will provide a means to assess at what length scales the electrode’s presence influences the IL. Moreover, by combining both bulk-phase and interfacial studies, tunability and optimization can be made possible.

## Supporting Information Available

Complete 360° video of Figure 6b (MP4)
Complete 360° video of Figure 8a (MP4)
Complete 360° video of Figure 8b (MP4)
Summary of simulation conditions; force field benchmarks; density as function of temperature and domain size; structure factor temperature-dependence; structure factor peak location comparisons at 300 K; partial structure factor partitioning; charge-correlation structure factor and relevant RDFs; carbon–carbon atomistic RDF (PDF)
All relevant OpenMM force field files and run scripts necessary to minimally reproduce MD simulations (ZIP)
