# Supplementary material for: Structural Properties of [N1888][TFSI] Ionic Liquid: A Small Angle Neutron Scattering and Polarizable Molecular Dynamics Study
Source: J Phys Chem B. 2024 Nov 5;128(45):11313–27. doi: 10.1021/acs.jpcb.4c06255 (PMC11571223; doi:10.1021/acs.jpcb.4c06255)
Supplement: Supplementary file 4 — jp4c06255_si_004.pdf [file jp4c06255_si_004.pdf]

Supporting Information for "Structural Properties of [N1888][TFSI] Ionic Liquid: A Small Angle Neutron Scattering and Polarizable Molecular Dynamics Study"

Shehan M. Parmar,[†] William Dean,[‡] Changwoo Do,[¶] James F. Browning,[¶] Jeffrey M. Klein,[§] Burcu E. Gurkan,[‡] and Jesse G. McDaniel[*,†]

†*Department of Chemistry and Biochemistry, Georgia Institute of Technology, Atlanta, GA, 30332, USA*

‡*Chemical and Biomolecular Engineering Department, Case Western Reserve University, Cleveland, Ohio, 44106, USA*

¶*Neutron Scattering Division, Oak Ridge National Laboratory, Oak Ridge, TN, 37831, USA*

§*MPA-11: Materials Synthesis and Integrated Devices, Los Alamos National Laboratory, Los Alamos, NM, 87545, USA*

* E-mail: mcdaniel@gatech.edu

Contents

S1 Matrix of all Simulation Conditions **S3**

S2 Force Field Benchmarks **S4**

S3 Density as function of temperature and domain size **S5**

S4 Structure Factor Temperature-dependence **S8**

S5 Structure Factor Peak Location Comparisons at 300 K **S8**

S6 Partial Structure Factor Partitioning **S8**

S7 Charge-correlation Structure Factor **S9**

S8 Carbon-carbon Atomistic RDF **S10**

S1 Matrix of all Simulation Conditions

Table S1: Summary of simulation conditions for corresponding analyses.

Ion Pairs	Temperature [K]	Total Simulation Time [ns]	ns/GPU hr	Property	Figure Numbers
200	300	300	1.42	S_{ZZ}, E_{coh}, density	2, S2, S3
	350	60	1.49	E_{coh}, density	S2, S3
	400	60	1.58	E_{coh}, density	S2, S3
	450	60	1.60	density	S3
	500	60	1.58	density	S3
900	300	300	0.33	S_{ZZ}, density	2, S3
	400	80	0.36	density	S3
1600	300	966	0.17	S_{ZZ}, S_{NN}, $g(r)$, CDFs, SDFs, $P(d)$, density	2, 4-10, S1, S3-S5
	350	100	0.18	density	S3
	400	200	0.19	S_{NN}, density	S3-S5
	450	100	0.19	S_{NN}, density	S3-S5
	500	100	0.19	S_{NN}, density	S3-S5

In Table S1, we summarize the simulation conditions for the corresponding property calculations. The total simulation times were chosen based on a tradeoff between computational complexity and desired property–i.e., properties such as density required less production simulation time than structure factor calculations. The computational complexity is highlighted in ns/GPU hours, where the primary simulation used in this work, 1600 ion pairs at 300 K, required ~ 10^3 GPU hours.

For simulations longer than 200 ns, total simulation time was acquired by running multiple simulations in parallel using equally-spaced trajectories from an equilibration simulation. Concretely, the 1600 ion pair system was first equilibrated at 500 K for 100 ns in the NPT ensemble. Equally-spaced trajectory frames from the equilibrated, 500 K system were used as initial starting trajectories for subsequent 1 K/ns annealing simulations down to 300 K. Annealing was then followed by independent, ~ 100−125 ns simulations to ultimately acquire the cumulative simulation times provided throughout Table S1.

S2 Force Field Benchmarks

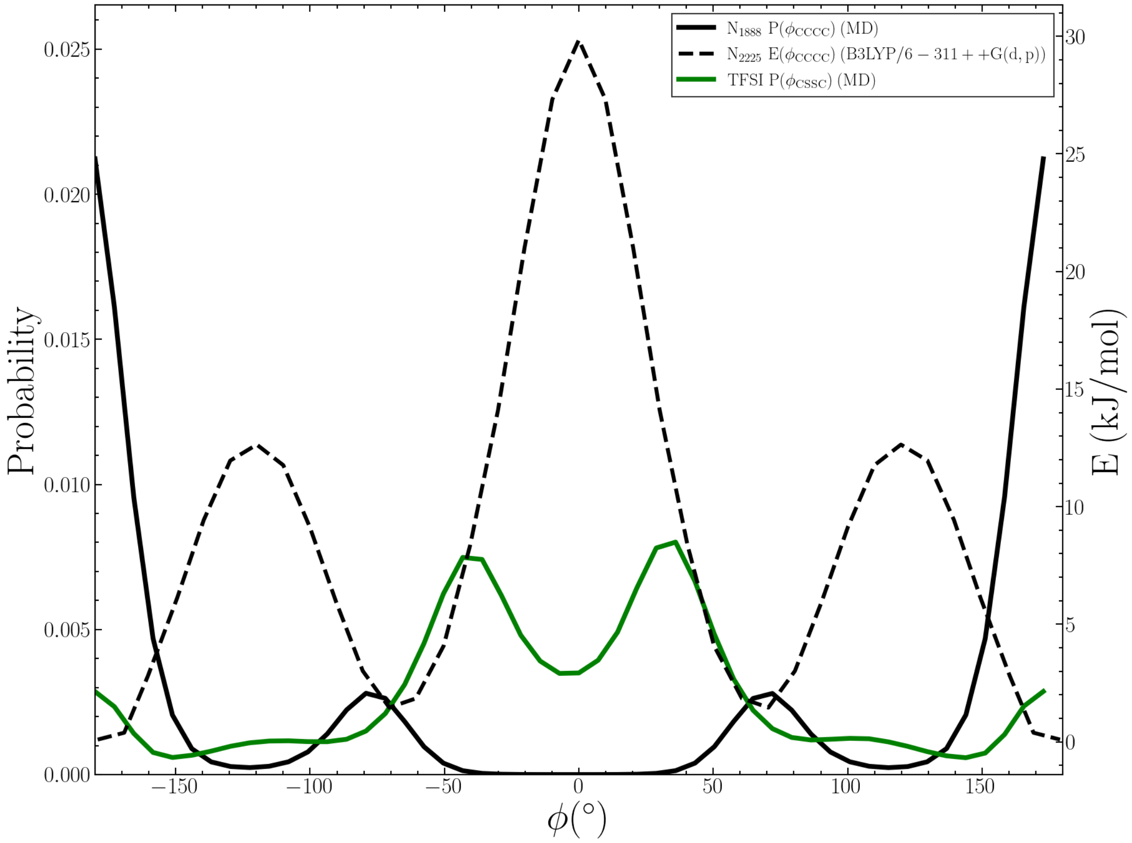

Figure S1: Dihedral distribution functions (DDFs) for (solid black) all C−C−C−C dihedral angles present in N_{1888} and (solid green) "pseudo" C−S−S−S dihedral angles present in TFSI. Quantum mechanical (dashed black) potential energy scan of C−C−C−C dihedral angle in N_{2225} from Ghatee and Bahrami[106] provided for qualitative comparison.

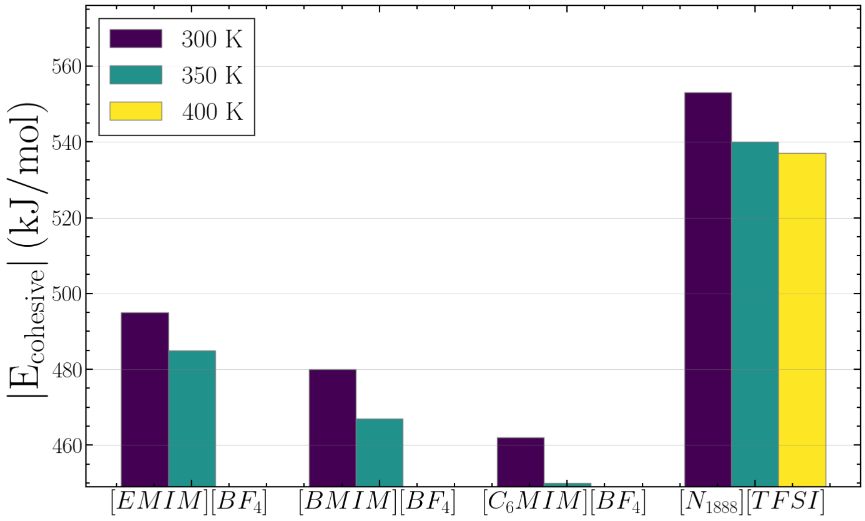

Figure S2: Liquid cohesive energies of $[N_{1888}]$[TFSI] compared to commonly studied ILs.[99]

S3 Density as function of temperature and domain size

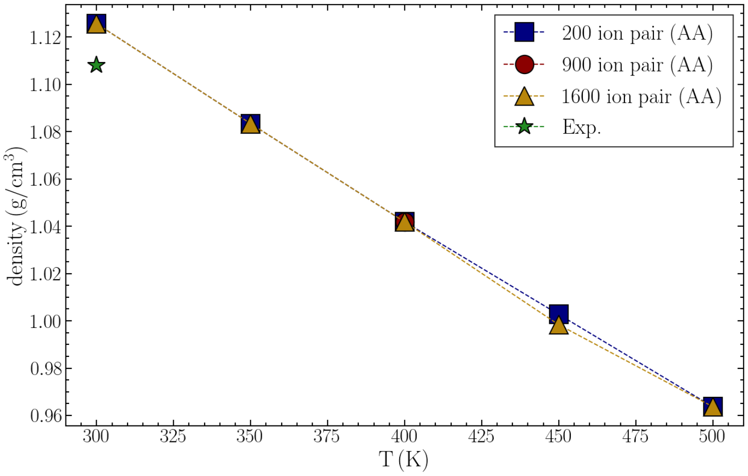

Figure S3: Density as a function of temperature at varied system sizes

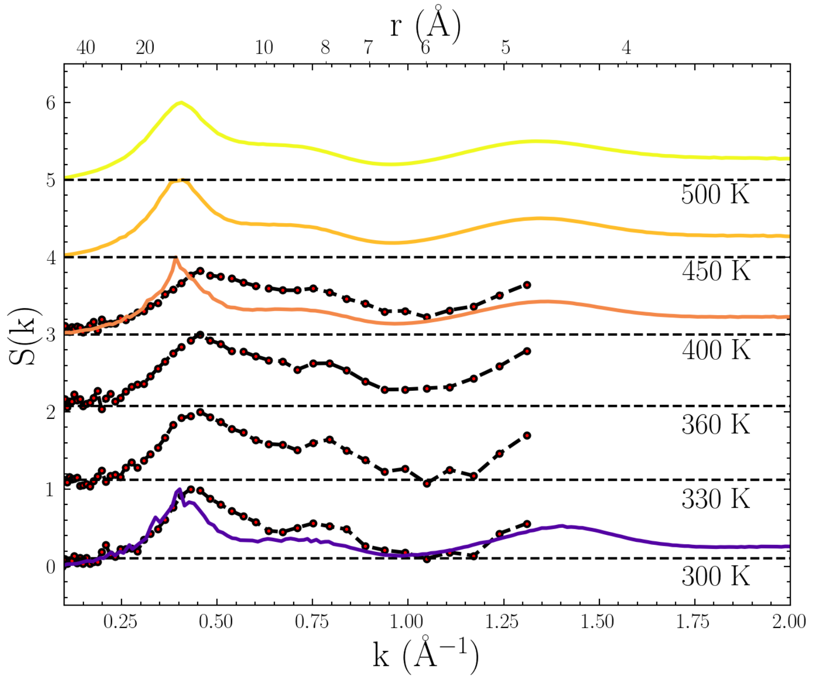

(a)

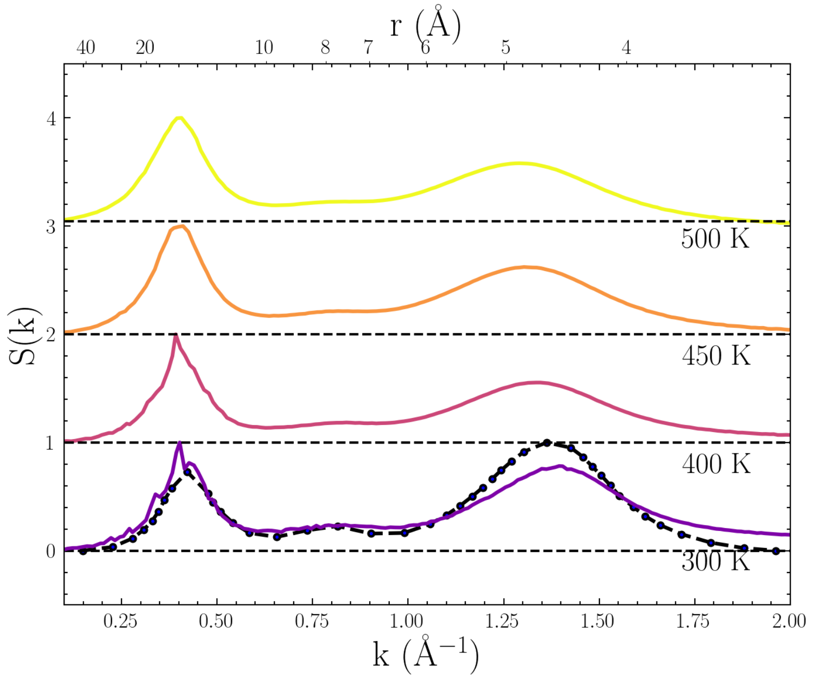

(b)

Figure S4: Measured SANS (S4a) and SAXS (S4b) and simulated structure factors for $[N_{1888}]$[TFSI] at four temperatures each. Red (S4a) and blue (S4b) circles are measured SANS/SAXS data and smooth lines are calculated from MD simulations.

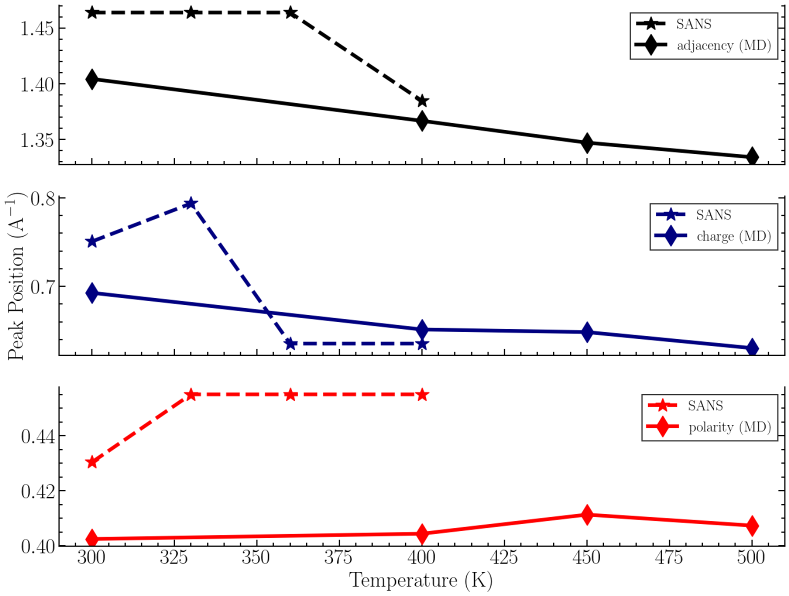

(a)

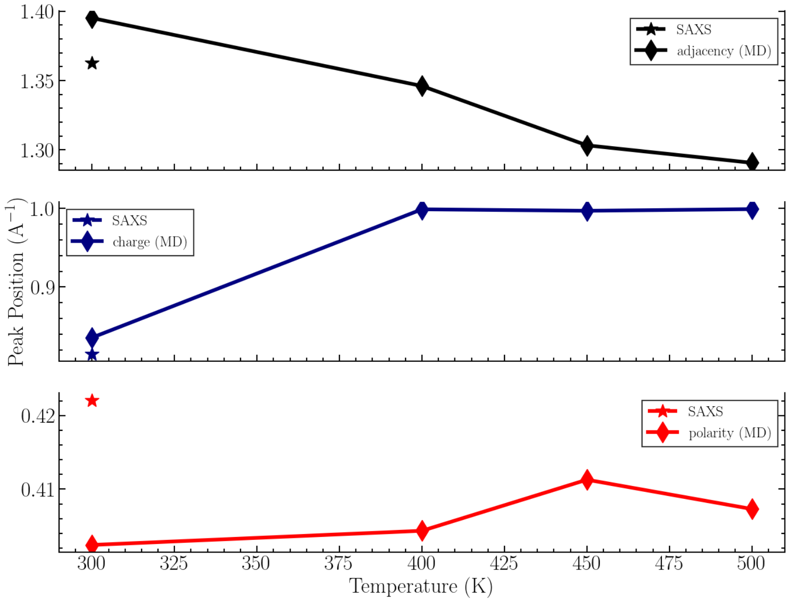

(b)

Figure S5: Temperature-dependence of peak location for neutron (S5a) and x-ray (S5b) scattering structure factors from Fig. S4. Simulated structure factors are shown as diamonds and experimental measurements are as stars.

S4 Structure Factor Temperature-dependence

S5 Structure Factor Peak Location Comparisons at 300 K

Table S2: Experimental and simulation comparison of peak locations for respective domains in scattering structure factors at 300 K.

**S(k)**	**Domain**	**SAXS/SANS** (Å^{-1})	**MD** (Å^{-1})	Δ%
X-ray	Polarity	0.422	0.402	4.76
	Charge	0.815	0.836	2.51
	Adjacency	1.363	1.395	2.36
Neutron	Polarity	0.430	0.402	6.51
	Charge	0.751	0.693	7.72
	Adjacency	1.464	1.404	4.10

S6 Partial Structure Factor Partitioning

The polarity domain is partitioned based on the polar and apolar contributions, where the total structural factor is decomposed into

$$S_{NN}(\mathbf{k}) = S^{p-p}(\mathbf{k}) + S^{ap-ap}(\mathbf{k}) + S^{p-ap}(\mathbf{k}), \tag{S1}$$

where the polar-polar, apolar-apolar, and polar-apolar components follow previous work,[93]

$$S^{p-p}(\mathbf{k}) = S^{an-an}(\mathbf{k}) + S^{cat_{head}-cat_{head}}(\mathbf{k}) + S^{cat_{head}-an}(\mathbf{k}) + S^{an-cat_{head}}(\mathbf{k}), \tag{S2}$$

$$S^{ap-ap}(\mathbf{k}) = S^{cat_{tail}-cat_{tail}}(\mathbf{k}), \tag{S3}$$

$$S^{p-ap}(\mathbf{k}) = S^{cat_{head}-cat_{tail}}(\mathbf{k}) + S^{cat_{tail}-cat_{head}}(\mathbf{k}) + S^{cat_{tail}-an}(\mathbf{k}) + S^{an-cat_{tail}}(\mathbf{k}). \tag{S4}$$

The charge domain is partitioned based on the cation and anion contributions,

$$S_{NN}(\mathbf{k}) = S^{+/+}(\mathbf{k}) + S^{-/-}(\mathbf{k}) + S^{+/-}(\mathbf{k}) + S^{-/+}(\mathbf{k}), \tag{S5}$$

where the adjacency domain's intermolecular and intramolecular components arise from

$$S^{+/+}(\mathbf{k}) = S^{+/+,intra}(\mathbf{k}) + S^{+/+,inter}(\mathbf{k}), \tag{S6}$$

$$S^{-/-}(\mathbf{k}) = S^{-/-,intra}(\mathbf{k}) + S^{-/-,inter}(\mathbf{k}). \tag{S7}$$

S7 Charge-correlation Structure Factor

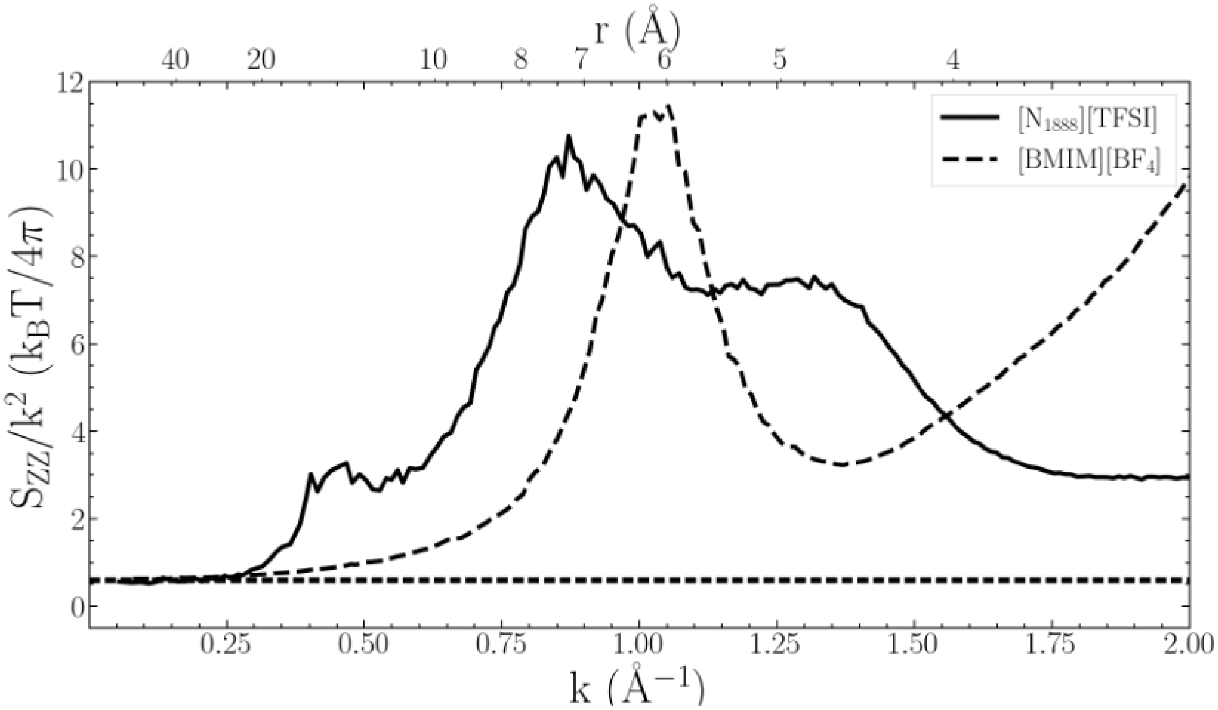

(a)

Figure S6: Charge-correlation structure factor comparison between $[N_{1888}]$[TFSI] and [BMIM]$[BF_4]$

S8 Carbon-carbon Atomistic RDF

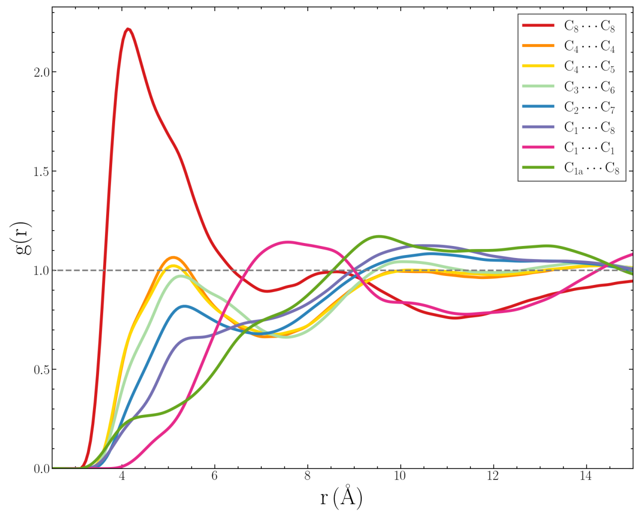

Figure S7: Carbon-carbon radial distribution functions, where C_1, C_2, C_3, C_4, C_5, and C_8 are carbon atoms of the octyl chain number in order of closest (C_1) to furthest (C_8) from the nitrogen center. C_{1a} follows the same notation as Santos et al.[80] for the N-methyl carbon.
